# Topographic organization in the olfactory bulb

**DOI:** 10.1007/s00441-020-03348-w

**Published:** 2021-01-06

**Authors:** Claudia Lodovichi

**Affiliations:** Neuroscience Institute CNR, Department of Biomedical Science, Veneto Institute of Molecular Medicine, Padova Neuroscience Center, Padova, Italy

**Keywords:** Olfactory bulb, Neuronal circuits, Topographic map, Odorant receptor, Electrical activity

## Abstract

The ability of the olfactory system to detect and discriminate a broad spectrum of odor molecules with extraordinary sensitivity relies on a wide range of odorant receptors and on the distinct architecture of neuronal circuits in olfactory brain areas. More than 1000 odorant receptors, distributed almost randomly in the olfactory epithelium, are plotted out in two mirror-symmetric maps of glomeruli in the olfactory bulb, the first relay station of the olfactory system. How does such a precise spatial arrangement of glomeruli emerge from a random distribution of receptor neurons? Remarkably, the identity of odorant receptors defines not only the molecular receptive range of sensory neurons but also their glomerular target. Despite their key role, odorant receptors are not the only determinant, since the specificity of neuronal connections emerges from a complex interplay between several molecular cues and electrical activity. This review provides an overview of the mechanisms underlying olfactory circuit formation. In particular, recent findings on the role of odorant receptors in regulating axon targeting and of spontaneous activity in the development and maintenance of synaptic connections are discussed.

## Introduction

The specificity of synaptic connections among neurons is essential to transform the electrical activity into meaningful neuronal codes. In most sensory modalities, nearby receptor neurons in the periphery project to nearby neurons in the target area, thereby maintaining spatial order. This spatial segregation of sensory afferents results in a “continuous” topographic map that encodes the quality, the intensity, and the location of sensory stimuli. In this paradigm, distinct features of sensory stimuli are analyzed according to the spatial distribution of receptor neurons in the periphery. This spatial pattern is faithfully maintained in higher brain areas where sensory information is processed to provide an internal representation of the external world (Kaas 1997; Feldheim and O'Leary 2010).

The olfactory system differs from this organizational plan, in several ways. In the peripheral structure, i.e., the olfactory epithelium, receptor neurons are almost randomly distributed. Spatial order is achieved in the olfactory bulb (OB), the first olfactory brain area, where sensory neurons expressing the same odorant receptor (OR) converge with exquisite precision to form glomeruli in invariant locations, resulting in the topographic map of the olfactory bulb (Mombaerts et al. 1996; Mombaerts 2001). In this case, the identity of the OR instructs the topography of the bulb, which results in thousands of discrete units, i.e., glomeruli. This spatial segregation of sensory afferents provides a “discrete” sensory map, where the quality and intensity of odor stimuli are encoded. Noteworthy**, **olfactory sensory neurons (OSNs) regenerate throughout life and constantly reform precise synaptic connections with the target field (Shepherd 2004). How does a highly spatial organization in the OB emerge from a random distribution of receptor neurons in the periphery? Compelling evidence indicates that ORs are involved not only in odor detection but also in the formation of the sensory map (Wang et al. 1998). Although ORs play a critical role in the OB topography, a complex interaction among ORs, other molecular cues, and electrical activity is required to carve the final configuration of the neuronal architecture of the OB.

This review provides an overview on olfactory circuit formation, with a focus on recent findings on the role of ORs and afferent spontaneous activity in the development and maintenance of neuronal circuits underlying the topography of the OB, in mice.

## Olfactory system organization: from the olfactory epithelium to the olfactory bulb

Odors are sensed by OSNs located in the olfactory epithelium that lines the posterior part of the nasal cavity. OSNs are bipolar neurons with a small soma and a single apical dendrite that ends in a swelling formation named “knob,” from which several thin filamentous structures, i.e., cilia, depart. ORs are expressed on the cilia that protrude in the nasal cavity, where they encounter odors carried by the airflow. From the opposite pole of the OSN soma, a thin unmyelinated, unbranched axon emerges and crosses the cribriform plate to reach the OB, the first olfactory brain area (Shepherd 2004).

ORs are seven transmembrane G-protein coupled receptors (Buck and Axel 1991) that upon binding odors activate specific olfactory G proteins, G_olf_, that stimulate adenylyl cyclase III (ACIII) to synthesize cAMP (Boekhoff and Breer 1990; Breer et al. 1990). Cyclic AMP then directly activates cyclic nucleotide-gated (CNG) channels, leading to an influx of Na^+^ and Ca^2+^ in sensory neurons (Liman and Buck 1994; Bradley et al. 2005). The rise of intracellular Ca^2+^ level opens Ca^2+^-activated-Cl^−^ channels in the ciliary membrane, resulting in an efflux of Cl^−^ that further depolarizes the cell to generate action potentials (Breer 1994; Menini 1995, 1999; Buck 1996; Prasad and Reed 1999; Reisert et al. 2005; Kaupp 2010).

The olfactory system has extraordinary discriminatory power, being able to detect a myriad of different odorant molecules, present in the environment even at very low concentrations. Such sophisticated discriminatory capacity relies on a wide repertoire of ORs and on a specific pattern of interaction between odors and ORs, named combinatorial code (Malnic et al. 1999). This code defines the ability of each OR to recognize multiple odors and of each odor to bind several ORs. Such complex interaction is made possible by distinct structural features in the odorant molecules, defined odotopes, that can interact with several ORs. In turn, each OR can recognize specific odotope in multiple odors (Malnic et al 1999). The specificity of the percept for a given odor is achieved by a unique combination of activated ORs. Given that the genome encodes more than 1000 ORs, this combinatorial receptor coding scheme allows the discrimination of a vast number of different odors.

## Topographic organization of the olfactory bulb

### The olfactory map

Unlike most sensory systems, the peripheral sheet of the olfactory system, i.e., the olfactory epithelium, exhibits a coarse topographic organization. Each OSN expresses only one in a repertoire of more than 1000 OR genes. The expression of a single type of OR is assured by the monoallelic activation of OR genes (Chess et al. 1994). Furthermore, it was reported that immature OSNs that express a given OR can switch expression of OR gene, although at low frequency. In contrast, immature neurons that express a defective OR switch OR gene expression with much higher probability. Once OSNs express a functional OR, this choice remains stable for the entire life cycle of the cell. This model suggests that the expression of a functional receptor is likely to signal, via a negative-feedback regulation, the termination of the OR switching process. Along with the monoallelic activation of OR genes, this mechanism contributes to ensuring that each OSN expresses a single functional OR for the entire life of the cell (Serizawa et al. 2003; Lewcock and Reed 2004; Shykind et al. 2004).

**Fig. 1:**
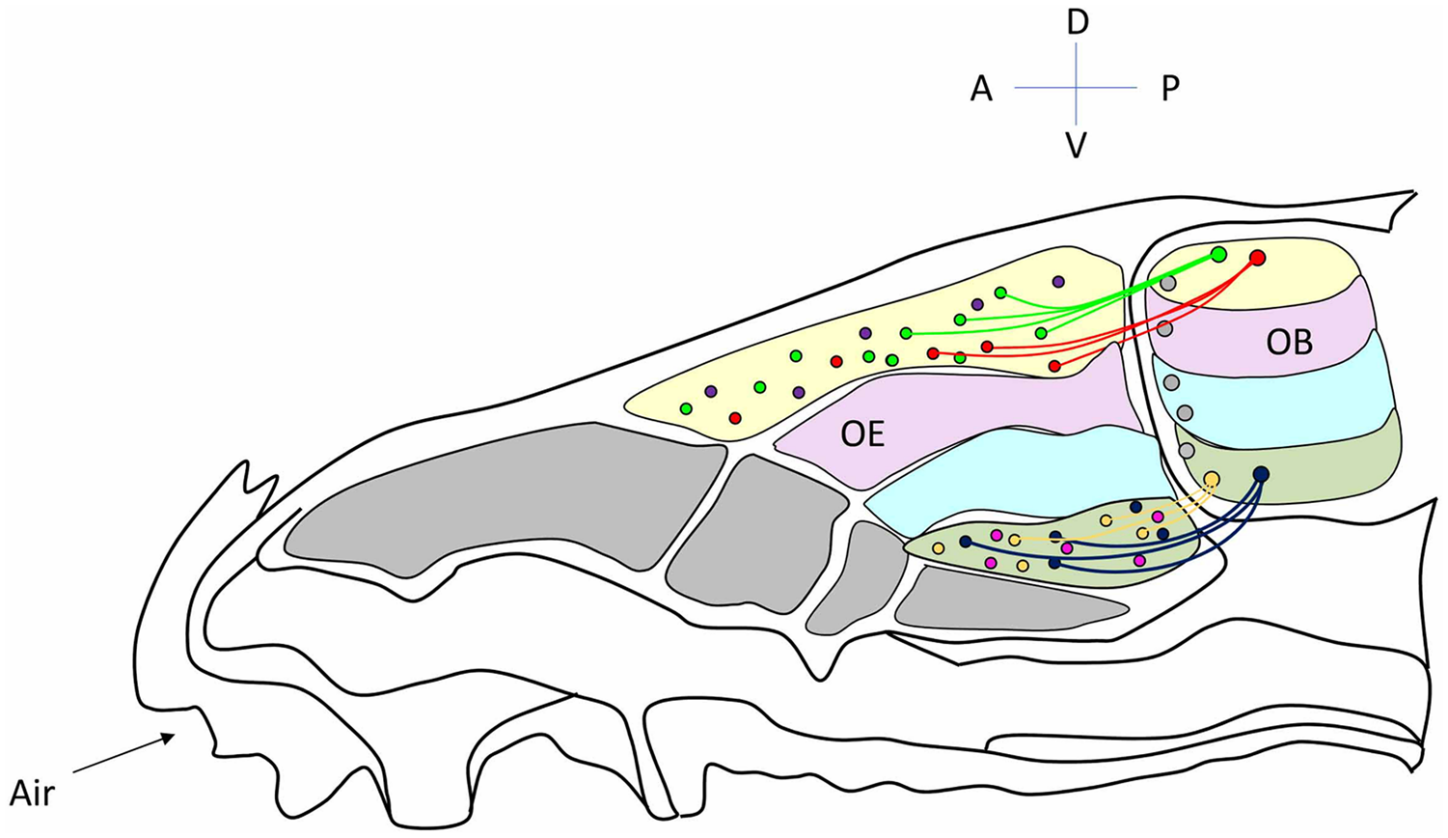
From the olfactory epithelium to the olfactory bulb. Schematic of the subdivision of the olfactory epithelium (*OE*) in zones along the dorso-ventral axis and the corresponding zones in the olfactory bulb (*OB*). Areas of different colors indicate the different zones. In the OE, within each zone, olfactory sensory neurons (*OSNs*) expressing different odorant receptors (*ORs*), indicated by circles in different colors, are almost randomly distributed. OSNs expressing the same OR converge to form glomeruli in specific locations of the OB. The distribution of OSNs in zones along the dorso-ventral axis of the OE is reflected in the location of the related glomeruli in corresponding zones along the dorso-ventral axis of the OB. Indeed, ONSs located in the most dorsal zone of the OE, project and converge to form glomeruli in the most dorsal area of the bulb. OSNs located in the most ventral area of the OE, project to the most ventral part of the  OB. OSNs which occupy intermediate positions in the OE, project in corresponding zones in the OB, along the dorso-ventral axis. For simplicity, only projections of OSNs located in the most dorsal and ventral areas, respectively, are shown. *D* dorsal, *V* ventral, *A* anterior, *P* posterior

OSNs expressing the same OR are confined within one of the large and overlapping zones in which the OE results divided along the dorso-ventral axis. Within the same zone, OSNs expressing different receptors are randomly interspersed (Fig. 1). The zonal organization of the epithelium resulted from the molecular characterization of OR expression by in situ hybridization (Ressler et al. 1993; Vassar et al. 1993; Miyamichi et al. 2005), and it still unclear what restricts the expression of a given OR in a specific zone.

**Fig. 2:**
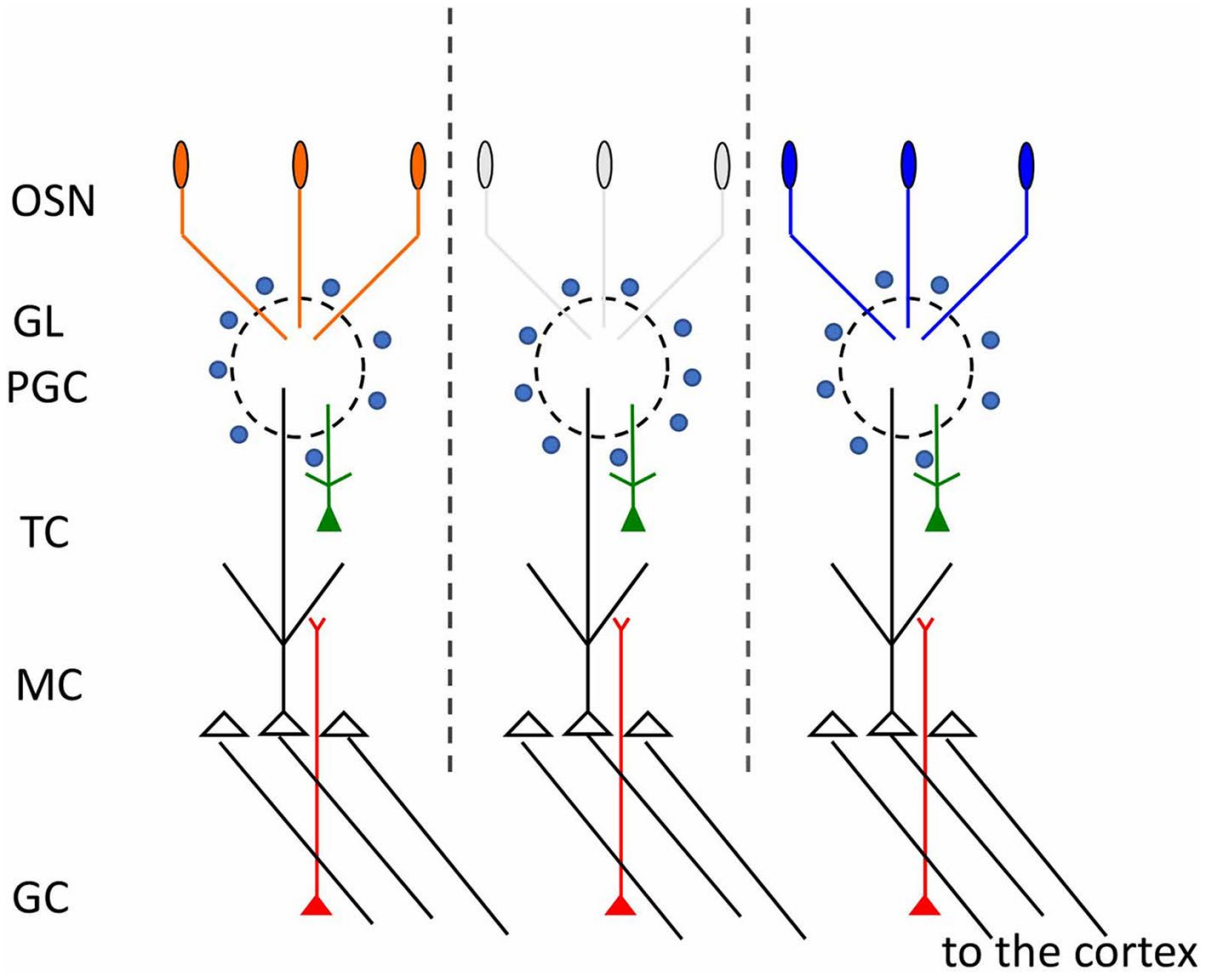
Odor columns in the olfactory bulb. Schematic diagram of the connectivity between pre- and postsynaptic cells in the olfactory bulb. Each glomerulus defines a functional unit, indicated also as odor column (depicted between the dashed vertical lines). *OSN* olfactory sensory neurons, *GL* glomerular layer, *PGC* periglomerular cells, *TC* tufted cells, *MC* mitral cells, *GC* granule cells

From an almost random distribution of ORs in the OE, spatial order emerges in the OB by the convergence of OSN axons expressing the same OR to form glomeruli in specific locations on the medial and on the later side of each OB (Fig. 1) (Ressler et al. 1994; Vassar et al. 1994). Glomeruli are spherical structures of neuropil that upholster the superficial layer of the OB. In each glomerulus, OSN axons make synapses with the postsynaptic cells of the OB, namely periglomerular cells, a heterogeneous population of cells that modulate the incoming sensory information (Aungst et al. 2003; Kosaka and Kosaka 2005; Wachowiak and Shipley 2006; Eyre et al. 2008) and with mitral and tufted cells, the output neurons of the OB (Shepherd 2004; Nagayama et al. 2010; Fukunaga et al. 2012; Igarashi et al. 2012). Remarkably, mitral and tufted cells extend their single apical dendrite exclusively in one glomerulus. Because of this pattern of pre- and post-synaptic connections, a glomerulus defines a functional unit that by analogy with the ocular dominance and orientation columns of the visual cortex is named “odor column” (Fig. 2). A glomerulus is therefore associated with a single OR as each OSN is associated with a single type of OR, from which the rules “one OSN, one OR and one glomerulus, one OR.” As a consequence of this pattern of synaptic connectivity, each odor column processes sensory information carried by a given OR (Shepherd 2004). Noteworthy, OSNs regenerate throughout life and continuously project with exquisite precision to form glomeruli in reproducible locations of the bulb (Graziadei and Monti Graziadei 1983; Hinds et al. 1984; Schwob 2002). The formation of glomeruli is a stepwise process. At first, OSNs project to a restricted area of the OB where they innervate several glomeruli, that result to be formed by fibers expressing different ORs, defined heterogeneous glomeruli. Subsequently, sensory axons undergo a remodeling and retracting process that leads to the formation of glomeruli formed exclusively by axons expressing the same OR, defined homogeneous glomeruli, a hallmark of the mature sensory map (Royal and Key 1999; Treloar et al. 1999). Glomerular maturation proceeds along different time courses for OSN axons expressing different ORs. Some, such as P2 glomeruli, are mature (i.e., homogeneous) at birth, while others, such as M72 and M71 glomeruli, reach a mature organization in postnatal life (Potter et al. 2001). Naris occlusion was reported to hamper the maturational process of M72 and M71 glomeruli. In absence of sensory inputs, the additional heterogeneous glomeruli persist (Zou et al. 2004), suggesting that sensory stimuli are required for glomeruli refinement. The postsynaptic partners of OSNs, the mitral cells, undergo a similar refinement process. Initially, mitral cells extend their multiple dendrites in numerous glomeruli. During postnatal development, these dendrites are gradually retracted until mitral cells exhibit a single apical dendrite that innervates a single glomerulus (Malun and Brunjes 1996).

The glomerular organization of the OB was unraveled by the molecular characterization of the spatial distribution of the OSN axonal projections, by in situ hybridization (Ressler et al. 1994; Vassar et al. 1994) and was directly “visualized” by an elegant genetic approach, devised by Mombaerts. In this seminal work (Mombaerts et al. 1996), a given OR sequence was genetically modified to be co-expressed with a reporter gene encoding tauLacZ. This genetic manipulation allowed the visualization of axons of OSNs expressing a given OR as they converge to form glomeruli in the OB. In a subsequent refinement of this genetic approach, tau-lac Z was replaced by GFP, making possible the visualization of the convergence of like-axons in vivo (Gogos et al. 2000).

As the spatial distribution of glomeruli expressing the same OR is invariant in the 2 OB of the same animal and among the OBs of different animals, it provides a proper topographic map. Detailed analyses of the glomerular array among different animals show that glomeruli expressing a given OR are located in a small subregion of the bulb, within this area glomerular position could exhibit subtle variations. Altogether, these findings corroborate a reliable placement and notably precise layout of glomeruli, which support the topography of the bulb (Strotmann et al. 2000; Schaefer et al. 2001; Soucy et al. 2009) ( but see (Oka et al. 2006).

As a consequence of the architecture of the OB topography and the combinatorial code that support the interaction between odors and ORs, an odor is encoded by a spatial pattern of activated glomeruli (Rubin and Katz 1999; Uchida et al. 2000; Belluscio and Katz 2001; Wachowiak and Cohen 2001; Soucy et al. 2009). The specificity of neuronal connections within the OB is therefore essential for proper information processing. Alterations in number, organization, and location of glomeruli result in deficits in odor discrimination (Lorenzon et al. 2015) (Fleischmann et al. 2008).

### The second level of topography in the olfactory bulb: the link between homologous glomeruli

**Fig. 3:**
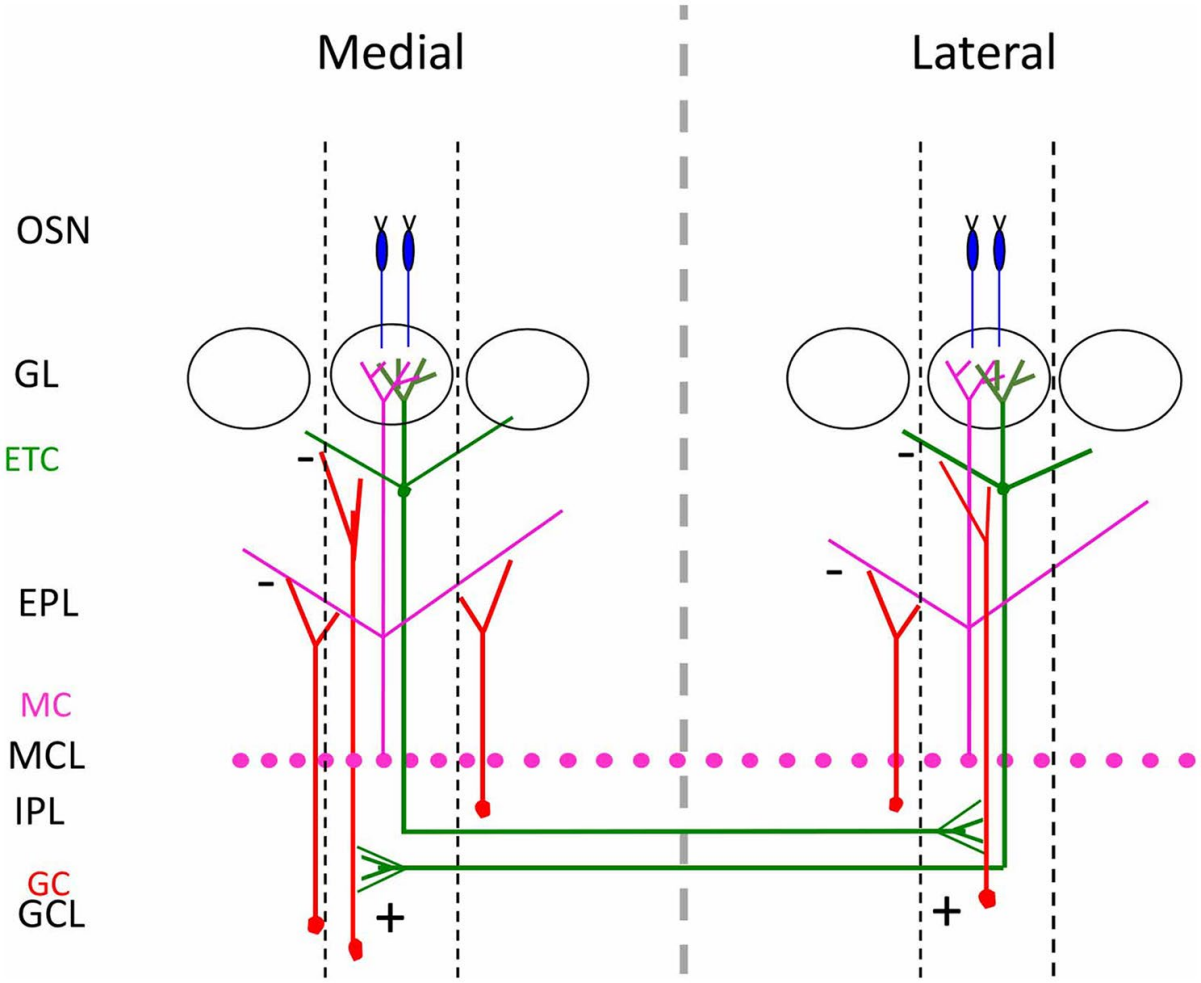
Link between homologous glomeruli. Schematic diagram of the intrabulbar link between homologous glomeruli. *OSN* olfactory sensory neurons, *GL* glomerular layer, *ETC* external tufted cells, *EPL* external plexiform layer, *MC* mitral cells, *MCL* mitral cell layer, *IPL* internal plexiform layer, *GC* granule cells, *GCL* granule cell layer

The convergence of OSN axons expressing the same OR to form a glomerulus in a specific location on the medial side and another glomerulus on the lateral side of each OB provides two mirrors symmetric maps of isofunctional glomeruli, also defined as homologous glomeruli. The exact anatomical structures and connectivity of these two maps as its role in odor information coding have remained enigmatic for a long time. Previous works (Schoenfeld et al. 1985) revealed that external tufted cells (ETCs) on the medial and on the lateral side of each OB reciprocally project to the internal plexiform layer, a band of tissue above the granule cell layer. The granule cells, in turn, form synapses on ETC projections. Since at the time, ORs had not been cloned yet, it was not possible to establish which anatomical structures were connected by the ETC projections. This conundrum was solved several years later. By performing dye injections confined to distinct GFP-labeled glomeruli and combining imaging of intrinsic signal with dye injections in the glomeruli activated within the functional maps, it was demonstrated that ETC projection links in a specific and reciprocal way homologous glomeruli (Belluscio et al. 2002; Lodovichi et al. 2003). Namely, ETC connected to the medial glomerulus project their axons in a restricted region of the inner plexiform layer on the opposite side of the OB, just beneath the homologous lateral glomerulus. In the inner plexiform layer, ETC axons form excitatory synapses with the dendrites of granule cells connected to the homologous lateral glomerulus. This inhibitory link is reciprocal (Fig. 3). These data reveal that this inhibitory connection links homologous odor columns in the two mirror-symmetric maps much as horizontal connections link iso-orientation columns in the primary visual cortex (Gilbert et al. 1990; Gilbert 1992). The intrabulbar link is present in the two OB of the same animal and in different animals, supporting the second level of topography in the OB.

The role of homologous glomeruli connected by the intrabulbar link in coding sensory inputs remains largely to be understood. It has been proposed that ETC can elicit slow modulatory action on mitral cells firing, which can be inhibitory or excitatory according to the timing of ETC activation with respect to the excitation of mitral cells by sensory stimuli. This evidence suggested that the intrabulbar link could shape the OB output via intraglomerular modulation of the firing pattern of mitral cells (Zhou and Belluscio 2008). This work provided important insight on neuronal dynamics within and between glomeruli connected by the intrabulbar link. However, the role of homologous glomeruli in odor coding and their impact on odor discrimination remain unaddressed questions, highlighting the need for further investigations. It is tempting to speculate that the intrabulbar link could elicit an oscillatory pattern of activity between the complement of mitral and tufted cells connected to the homologous glomeruli, since reciprocal inhibitory interactions are a hallmark of oscillations, in several systems. In this respect, it is worth noticing that odor-induced oscillations were shown to contribute to odor discrimination in several species (Stopfer et al. 1997; Friedrich and Stopfer 2001; Laurent 2002; Kay et al. 2009). Whether the intrabulbar link could trigger oscillatory activity, adding a temporal dimension to the spatial pattern of activated glomeruli, to refine and empower odor discrimination, remains to be clarified.

## Role of the odorant receptor in the formation of the sensory map

The tight link between the OR identity and the specific neuronal wiring underlying the topography of the OB prompted to hypothesize that the OR plays a role not only in odor detection but also in axon targeting. The latter was demonstrated by a series of elegant genetic experiments (Wang et al. 1998). In this seminal work, they found that deletion or nonsense mutations in OR sequence, namely in the P2 coding gene, caused P2 axons to wander in the OB. Subsequently, it was demonstrated that upon deletion of the endogenous OR gene, the same OSNs expressed a different OR and therefore target different glomeruli (Serizawa et al. 2003; Lewcock and Reed 2004; Shykind et al. 2004). In the same study, Wang and collaborators (1998) performed a series of receptor substitution experiments that replace the coding region of a given OR, such as P2, with the coding region of another OR, such as M71. Axons expressing M71 in the locus of the P2 gene project to a location that was different from either the position of the endogenous P2 glomerulus and the endogenous M71 glomerulus. The position of the “replaced” or “swapped” glomeruli, with respect to the location of the endogenous glomeruli, was shown to depend on several factors such as (1) the degree of homology between the swapped OR sequences, (2) the location in the olfactory epithelium zone of the 2 swapped receptors, and (3) the chromosomal location of the swapped coding regions. However, even when receptor substitution experiments replace the coding region of P2 with the coding region of P3, two OR genes that share a high degree of homology, are expressed on the same chromosome, and in OSNs located within the same olfactory epithelium zone, P3-P2 axons converge in the proximity of the endogenous P3 glomeruli, but sill in a different location in respect to the endogenous P3 glomerulus. Altogether, these data demonstrated that the OR plays an instructive role in OSN axon targeting, although it is not the only critical factor, and other guidance cues contribute to defining glomerular locations in the OB. A series of receptor substitution experiments were performed also by Feinstein et al. (2004), in Mombaerts laboratory. The results were similar to the ones obtained by Wang and collaborators, with some important distinctions. Feinstein and Mombaerts (2004) performed receptor substitution experiments between two similar ORs, M71 and M72. By swapping a portion of the coding region between M71 and M72, they identified a “core” region in the OR coding sequence that was sufficient and necessary to define the OR identity and direct axons to their proper target. In addition, they found that the level of OR expression was important in directing OSN axons to their glomeruli, as a drastic reduction of OR level resulted in aberrant sensory axonal projections. Interestingly, they found that replacing the coding region of the beta-adrenergic receptor into the locus of an OR receptor (i.e., M71) results in sensory axons coalescence into glomeruli on the medial and the lateral side of each OB. By contrast, when the V1R-type of the vomeronasal receptor, V1R2b (Rodriguez et al. 1999) was replaced in the locus of an OR, no glomerular formation was observed. The key difference between these two 7 transmembrane receptors is the intracellular signaling cascades coupled to them. Beta-adrenergic receptors can couple to G_s/olf_ protein (Jones and Reed 1989) leading to cAMP synthesis, while vomeronasal receptors V1R2b are not thought to couple to G_s/olf_ protein (Feinstein et al. 2004; Feinstein and Mombaerts 2004). These findings highlighted the importance of the OR-derived cAMP signal in the coalescence of like axons, a phenomenon that has been further corroborated by subsequent works (see below Imai et al 2006).

What remained to be clarified was the mechanism by which ORs could regulate axon targeting. Two hypotheses have been proposed. The first one postulates that in order to regulate axon targeting ORs should be expressed on the axon terminal-growth cone, a suitable location for putative axon guidance molecules. Following a classic paradigm of axon guidance, ORs expressed at the axon terminal recognize cues elaborated in the target brain region, i.e., the OB (Wang et al. 1998). The second hypothesis posits that ORs regulate axon targeting by homophylic interactions between like-axons (Feinstein and Mombaerts 2004). The two hypotheses are not necessarily mutually exclusive but could regulate different stages of the OSN convergence, as subsequent studies indicated. A few years after postulating it, the first hypothesis was corroborated by the finding that ORs are expressed and locally translated at the axon terminal (Barnea et al. 2004; Strotmann et al. 2004; Dubacq et al. 2009). Notably, ORs are expressed in the distal portion of the axons, while the soma and the proximal part of the axon are devoid of OR expression. The specific and exclusive location of ORs at the axon terminal and at the cilia suggested distinct roles of ORs in these two locations. The local translation and expression of ORs at the axon terminal support a direct role for ORs in the axon guidance process, in line with numerous evidence that indicates the axon terminal as an autonomous compartment where molecules that are implicated in axon guidance are locally translated and expressed. The major advantage of the axon terminal as an autonomous compartment that regulates locally protein translation and synthesis is to endow the axon with the ability to respond promptly to the cues encounter along the pathway towards its final target (Holt et al. 2019).

**Fig. 4:**
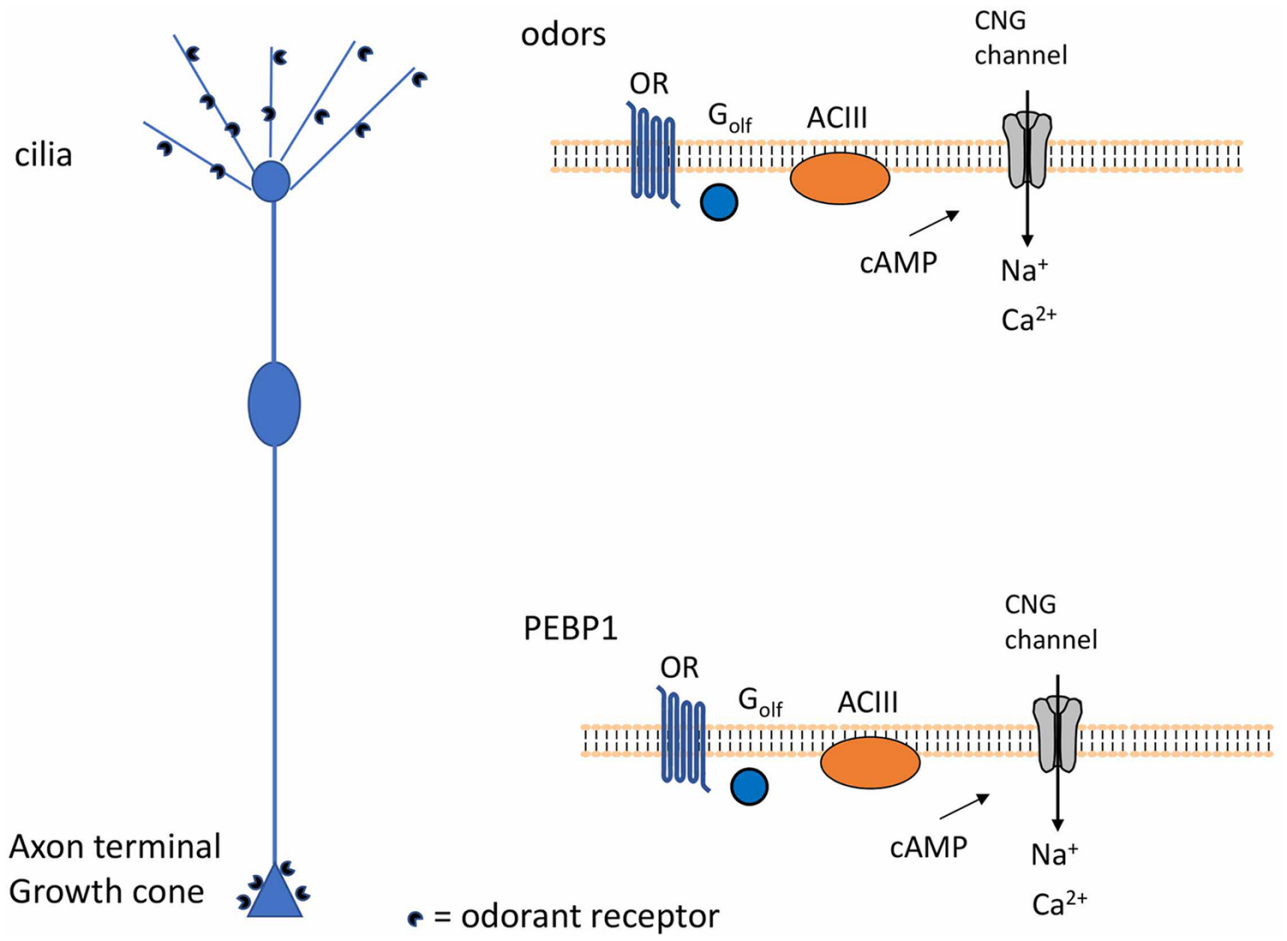
Localized cAMP and Ca^2+^ synthesis at the cilia and at the axon terminal of olfactory sensory neurons (OSNs). Odorant receptors are expressed in specific locations in OSNs: at the cilia and at the axon terminal. In both sites (i.e., cilia and axon terminal) ORs are coupled to localized increases of cAMP and Ca^2+^. *OR* odorant receptor, *ACIII* adenylyl cyclase III, *CNG* channels cyclic nucleotide gated channels

In order to establish a direct involvement of axonal ORs in the axon guidance process, it was critical to assess whether axonal ORs were functional and define the signaling pathway coupled to them. The first evidence in that direction came from the work of Maritan et al. (2009). By studying the spatio-temporal dynamics of cAMP and Ca^2+^ in isolated OSNs and ex vivo, they found that axonal ORs are activated by odors, the only known ligands of ORs at the time, and are coupled to cAMP synthesis and Ca^2+^ influx through CNG channels (Fig. 4). Furthermore, they showed that the selective odor activation of axonal ORs was followed by nuclear translocation of the protein kinase A (PKA) catalytic subunit. Altogether, these results indicate that axonal ORs are active and coupled to local increases of Ca^2+^ and cAMP (Fig. 4). These second messengers and in particular cAMP could exert its action locally, regulating growth cone steering, and also at the nuclear level, via PKA nuclear translocation. In this location, PKA could trigger the expression of genes encoding proteins involved in the axon guidance process (Maritan et al. 2009).

Cyclic AMP is not the only second messenger generated upon odor-OR activation, cGMP is also synthesized. Compared to cAMP dynamics, cGMP exhibits a slow and sustained rise. This kinetic suggested that cGMP may not be involved in the initial odor detection events but may regulate long-term cellular responses, including axon elongation (Kroner et al. 1996); (Zufall and Leinders-Zufall 1998). Origin and spatio-temporal dynamics of cGMP signaling were analyzed by performing real-time imaging in OSNs (Pietrobon et al. 2011). These experiments revealed that in response to odor OR activation, a rise in cGMP was observed at the cilia but also at the axon terminal-growth cone, where cGMP is locally synthesized. The odor OR-dependent rise of cGMP was due to soluble guanylyl cyclase (sGC) activation by nitric oxide and requires an increase in cAMP levels. The link between the OR-derived cAMP and nitric oxide was found to be the increase of Ca^2+^ concentration elicited by plasma membrane channels activation and/or release of Ca^2+^ from stores. Similarly to cAMP, cGMP has the potential to act locally, at the cilia and at the axon terminal, and also at the nuclear level, since also the rise of cGMP was found to be associated with nuclear CREB phosphorylation in vitro and in vivo (Pietrobon et al. 2011).

These results were of relevance for the putative role of axonal ORs as guidance molecules since cAMP, cGMP, and Ca^2+^ are known to exert a key role in the axon guidance process in several systems (Song et al. 1997; Nishiyama et al. 2003; Zheng and Poo 2007; Tojima et al. 2011; Akiyama et al. 2016) including the olfactory system (Imai et al. 2006). Among the second messengers, the OR-derived cAMP was found to exert a critical role in OSN axon targeting. To provide compelling evidence of the role of cAMP in axonal projection, Imai et al. generated a defective OR-I7, in which the tripeptide region (DRY) required for binding G-protein was mutated. As a consequence of this genetic manipulation, the defective OR was unable to couple to G protein and therefore to stimulate cAMP synthesis. Axons expressing the defective OR-I7 failed to converge to form glomeruli and remained in the nerve layer, never entering the glomerular layer. The absence of convergence of mutant OSNs could be ascribed to altered growth cone dynamics and/or altered expression of gene encoding axon guidance molecules, associated with the absence of cAMP synthesis. Imai and collaborators found that cAMP modulates the expression of axon guidance molecules, such as neuropilin-1 (Nrp1), that along with semaphoring-3A (Sema3A), the repulsive ligand of Nrp1, regulate the position of glomeruli along the antero-posterior axis in the OB (Imai et al. 2006, 2009; Sakano 2010). These results strengthened the importance of OR-derived cAMP in the convergence of like-axons but left open critical questions related to two important aspects: (1) the mechanism of activation of ORs that leads to cAMP rise and (2) the site of cAMP synthesis, namely whether axonal ORs are the origin of the OR-derived cAMP and are therefore directly involved in the sensory map formation.

## Identification of the first putative ligand of axonal odorant receptors

In the classic paradigm of axon guidance, cues elaborated in the target areas activate molecules concentrated on the axon terminal-growth cone, to govern axon direction. According to this model, axonal ORs should recognize molecules expressed in the OB. This hypothesis formulated more than 20 years ago (Wang et al. 1998) remained without direct evidence till recently, when Zamparo et al. (2019), through an unbiased screening of molecules expressed in the OB, identified a pool of proteins able to induce Ca^2+^ rise at OSN axon terminal, when locally applied. Further support that the Ca^2+^ rise was associated with OR activation, was obtained by transfecting HEK cells with specific ORs. Ca^2+^ rises in response to the active pool of molecules were observed only in HEK cells expressing specific ORs and not in HEK cells lacking the specific ORs. Finally, by mass spectrometry of the active pool of molecules, it was identified the first putative ligand of axonal ORs: phosphatidylethanolamine-binding protein 1 (PEBP1) (Zamparo et al. 2019). PEBP1 is a cytoplasmic protein of 21 kDa that can be secreted via a non-classic pathway. The small molecular weight, the ability to be secreted and modulate G protein-coupled receptors (i.e., beta-adrenergic receptor Goumon et al. 2004; Granovsky and Rosner 2008), and the presence of olfactory deficits in mice carrying a null mutation in PEBP1 (Theroux et al. 2007) make PEBP1 a prime candidate as a putative OR ligand. Local application of PEBP1 elicited Ca^2+^ rise in OSN axon terminals and in HEK cells transfected with specific ORs but not in HEK cells not expressing these specific ORs. The latter finding further corroborated that Ca^2+^ rise in response to PEBP1 was due to OR activation. However, since currently reliable binding assays to demonstrate that a given putative ligand physically interacts with OR are not available, it was not possible to demonstrate the direct binding of PEBP1 to the ORs. In this scenario, the activation of ORs in response to PEBP1 was inferred by the increased Ca^2+^ level in the stimulated OSNs or HEK cells. The rise of second messengers (cAMP and or Ca^2+^) is the classic readout of OR activation, adopted to ascertain the activation of ORs by their cognate odor-ligands (Malnic et al. 1999; Zhuang and Matsunami 2007). Generation of ORs with different affinities for PEBP1 could also contribute to deepening our understanding of the role of PEBP1 in axon targeting. Notably, among the OR tested, M72 was not responsive to PEBP1, suggesting the presence of additional ligands able to regulate axon guidance in OSN expressing different subsets of ORs.

The in vivo physiological relevance of PEBP1 was demonstrated by the fact that in mice carrying a null mutation for PEBP1 (PEBP1KO mice), the convergence of OSN axons expressing P2-OR was disrupted. P2 axons targeted not only the main glomerulus but also several additional adjacent glomeruli. Furthermore, the position of the P2 glomeruli was significantly shifted along the antero-posterior axis, albeit the location of P2 glomeruli along the dorso-ventral axis was unaffected (Zamparo et al. 2019). These results are in line with previous works that demonstrated that the position of glomeruli along the dorso-ventral axis is not dictated by the OR identity but reflects the location of the ORs in distinct zones of the epithelium (Cho et al. 2009) (and see below). Altogether, these findings indicate that PEBP1 is instrumental to provide OSNs with information to reach the proper location along the A-P axis.

The convergence of M72 expressing axons was not altered in PEBP1 KO mice, confirming that M72 is not responsive to PEBP1 and suggesting again (see above) the potential for additional ligands for modulating axon guidance for other subpopulations of OSNs. As for the expression pattern of PEBP1, it results in a global gradient along the antero-posterior axis. At the local level, however, glomeruli surrounded by periglomerular cells with a high level of PEBP1 expression were intermingled to glomeruli around which PEBP1 labeling could be hardly detected (Zamparo et al 2019). This spatial distribution of PEBP1 resembles the expression pattern of neuropilin-1, another molecule involved in the regulation of glomeruli position along the antero-posterior axis (Col et al. 2007; Assens et al. 2016 but see also Zapiec et al. 2016). The discrete expression pattern of molecules involved in the formation of the sensory map reflects the discrete nature of the topography of the olfactory bulb. In contrast, continuous gradients of guidance cues are found in continuous topographic maps, such as the visual map (Luo and Flanagan 2007).

As mentioned before, the observation that at least one receptor (M72) was not responsive to PEBP1, suggested the potential for additional ligands, whose identity and number remain currently unknown. As for the number of putative ligands, two scenarios can be envisioned. (1) The number of ligands equals the number of ORs. This model would imply more than one thousand cues, with a very circumscribed and specific pattern of expression in the OB, being each molecule able to activate a single OR. (2) There is a limited number of cues, expressed in gradients within the OB. The data collected by Zamparo et al. (2019) favors the second model. PEBP1 was reported to be broadly expressed, suggesting that it could interact with different types of ORs. This hypothesis was corroborated by the evidence that PEPB1 activates not only P2 but also EG, Olfr62, and S6 receptors. Each receptor exhibited a different degree of response, suggesting different affinity of each OR for PEBP1.

How can cues elaborated in the OB, such as PEBP1, direct OSNs to their final destination? The model proposed is that a few molecules expressed in gradients in the OB direct distinct subpopulation of ORs in specific sub-regions of the OB. Within these regions, characterized by a patchy distribution of PEBP1 (or other ligands), the different affinity of each OR for the specific ligand, such as PEBP1, defines the exact location for the convergence of like axons to form glomeruli (Zamparo et al. 2019).

PEBP1 was found to be expressed mostly in periglomerular cells, a suitable location for molecules that regulate axon targeting. The role of postsynaptic cells in the formation of the sensory map is in line with previous works in which it was reported that molecules synthesized in periglomerular and mitral cells, such as Eph and IGF1, interact with cues expressed in OSN axons (i.e., Ephrin and IGF1 receptor) to direct OSN axons to their glomeruli (Cutforth et al. 2003; Scolnick et al. 2008). Axon targeting in OB appears to recapitulate the classic paradigm of axon guidance in which cues on the axon terminal-growth cone of the projecting neurons are activated by complementary sets of cues elaborated in the target areas (Feldheim and O'Leary 2010). The set neuropilin-1/semaphorin-3A, which was reported to be expressed in OSNs (Imai et al. 2009), appears an exception in respect to this model. On the other hand, previous work indicated that postsynaptic cells appear dispensable for the formation of glomeruli (Bulfone et al. 1998). In mice bearing a homozygous null mutation in Tbr1/Tbr2 that lack most projecting neurons and in double knockout mice for Tbr1/Tbr2, lacking most GABAergic interneurons, the convergence of P2 axons to form glomeruli appeared normal (Bulfone et.al 1998). It is worth noticing that the double knockout mice die a few hours after birth, and exhibit dysmorphic OB, making it difficult, if possible, to ascertain the location of P2 glomeruli. Altogether, these data seem to indicate that postsynaptic cells are not required for the coalescence of like axons to form glomeruli, while they are important for directing axon targeting and define the unique location of glomeruli in the OB.

## Other molecules involved in the sensory map formation

Although ORs have an instructive role in the formation of the olfactory map, additional sets of molecules contribute to defining the position of glomeruli along the antero-posterior (A-P), dorso-ventral (D-V), and medio-lateral (M-L) axis, respectively. It is worth noticing that the identity of ORs is highly correlated and can regulate the expression of these additional axon guidance molecules. As described above, OR-derived cAMP regulates the expression of different levels of neuropilin-1 in different OSN axons, leading to a gradient of Nrp1 along the A-P axis, that in turn govern positioning of glomeruli along the same A-P axis (Imai et al. 2006, 2009). Mutations of neuropilin-1 or semaphorin-3A, its repulsive ligand, disrupt the arrangement of glomeruli formation along A-P (Schwarting et al. 2000, 2004; Taniguchi et al. 2003). The OR identity dictates the expression level of another classic family of guidance molecules: ephrins. Ephrins regulate axon targeting in several sensory modalities. In the visual system, for example, a medio-lateral gradient of EphA receptors on the axons of the retinal ganglion cells matches an overlapping gradient of two Ephrin ligands, i.e., ephrin-A5 and ephrin-A2, to regulate the spatial segregation of retinal ganglion cells axons to the brain (Luo and Flanagan 2007). In the olfactory system, the identity of the OR defines the type and the level of expression of guidance cues. OSN expressing different ORs contain different levels of ephrin-A3 and ephrin-A5 on their axons. The cognate receptors EphA3 and A5 are expressed in postsynaptic cells, in the OB. Deletion of ephrin-A5 and ephrin-A3 in OSNs causes a posterior shift in the position of the corresponding glomeruli, whereas overexpression of ephrin-A5 and ephrin-A3 leads to an anterior shift in glomeruli location (Cutforth et al. 2003). Both in the visual and in the olfactory system ephrin and Eph are expressed in pre- and postsynaptic neurons to favor the interaction of incoming sensory neurons with the target area and drive sensory axons to their proper destination. The pattern of expression of these guidance cues is however significantly different in the two systems, reflecting the continuous or the discrete nature of the topographic organization of these sensory modalities, respectively.

The convergence of sensory neurons along the M-L axis appears to be regulated by insulin-like growth factor (IGF) signaling. The complementary expression of IGF1 and IGF2 in the OB and IGF receptors on the axons of OSNs suggested a possible role of IGF signaling in the projection of OSN axons to the OB. This hypothesis was corroborated by the evidence that disruption of genes encoding IGF ligands or IGF receptors causes a severe misrouting of OSN axons away from the lateral aspect of the bulb, toward ventral and medial ectopic locations, resulting in a deep perturbation of the two mirror-symmetric maps that characterize the OB topography. This study indicated that IGF signaling is required for sensory innervation of the lateral side of the OB (Scolnick et al. 2008).

Unlike OSN projections along the A-P axis, spatial segregation of sensory axons along the D-V axis reflects the distribution of OSNs in zones in the epithelium. The correlation between the location of OSNs in different zones of the olfactory epithelium and the position of glomeruli in corresponding zones of the OB raises the possibility that the zonal location instructs OSNs to express molecular determinants that regulate axon elongation and targeting. According to this model, OSNs located in a given zone of the epithelium express specific types and levels of molecules that regulate axonal projections to the OB. Two sets of repulsive ligands/receptors, Slits/Robo2 and semaphorin-3F/neuropilin-2, have been reported to regulate OSN projections along the D-V axis. A dorso-ventral gradient of Robo2 receptors expressed in the axons of OSNs combined to a complementary pattern of chemorepellent ligand Slit in the OB direct the projections of OSNs confined in a given zone to the corresponding region in the bulb. Axons that normally target the dorsal region of the bulb are misrouted toward ventral ectopic locations of the bulb, in mice carrying a null mutation in Robo2 encoding gene. Ablation of Slit expression causes target defects similar to the ones observed in Robo 2 mutant mice, indicating that Robo2-Slit regulates the D-V segregation of sensory axons (Cho et al. 2007). Noteworthy, the secreted semaphorin-3F (sema3F) receptor, neuropilin-2, is expressed in a ventro-dorsal gradient, i.e., low expression in the dorsal part of the OE, and high expression in the ventral OE. Mutations in the genes encoding neuropilin-2 and/or its ligand semaphorin-3F demonstrate their role in regulating OSN projection along the D-V axis (Cloutier et al. 2002, 2004; Walz et al. 2002; Takeuchi et al. 2010).

The formation of the sensory map is a stepwise process, where a coarse layout of sensory projections (i.e., global targeting) is followed by the coalescence of like-axons to form glomeruli (i.e., local sorting). Several studies suggested that local axon sorting is mediated by adhesion molecules. Axons expressing the same set of adhesion molecules may fasciculate together, and interactions of different strength may be generated through the combinatorial expression of cell adhesion molecules. To seek to identify those molecules, Sakano and Axel laboratories focused on the identification of genes whose expression correlates with the OR identity. The expression of two homophilic adhesive molecules Kirrel 2 and Kirrel 3 (Serizawa et al. 2006), along with the already identified ephrin-A5/EphA5 (Cutforth et al. 2003), was found to be highly correlated to a given OR, in mice overexpressing that OR type. Furthermore, it was found that odor-evoked activity modulates the expression profile of those molecules, although in different directions. In CNG KO mice, Kirrel 2 and EphA5 were downregulated, while ephrin-A5 and Kirrel 3 were upregulated (Serizawa et al. 2006; Sakano 2010). OSN convergence analysis unraveled that the gain of function of those genes results in duplicated glomeruli. Yoshihara group identified another adhesive molecule, BIG2, that is expressed in sensory axons in OR specific manner, and favors axon sorting, acting on yet unidentified homophilic partners (Kaneko-Goto et al. 2008). More recently, it was shown that all three gene clusters encoding cell surface protocadherin proteins, *Pcdhα*,* Pcdhβ*, *Pcdhγ*, cooperate to provide OSN axons with a unique combination of cell surface molecules required to promote the coalescence of like-axon into glomeruli. A null mutation in a single *Pcdh* gene cluster results in subtle defects of axonal convergence, suggesting that the remaining two active gene clusters provide OSN with sufficient cell surface diversity to ensure almost normal coalescence and formation of protoglomeruli. When all three *Pcdh* clusters were deleted, axons converge approximately in the proper location in the OB, however failed to form protoglomeruli (Mountoufaris et al. 2017). Altogether, these results indicate that a set of adhesion/repulsive cues provide OSN axons with the cell surface diversity required to favor coalescence of like axons to form glomeruli.

## The odor-evoked activity does not significantly impact neuronal wiring in the olfactory bulb

The development of neuronal circuits is regulated by molecules expressed in specific spatio-temporal patterns and by electrical activity. To analyze the effect of odor-evoked activity in the development of the OB topography, several lines of transgenic mice carrying mutations in specific molecules of the OR signaling transduction pathway were generated. This genetic approach allowed the identification of the specific role of each molecular determinant of the transduction pathway.

Mice carrying a targeted mutation in the alfa subunit of the CNG channels (Lin et al. 2000), a key component of the OR signaling transduction pathway, failed to exhibit responses to a wide range of odorant stimuli. The convergence of like axons to form glomeruli in invariant locations in each bulb was only slightly perturbed in these mice (Lin et al. 2000). This was shown by analyzing the convergence of several different populations of OSNs. Subtle alterations of convergence were reported only for M72-expressing axons (Zheng et al. 2000). The effect of evoked activity on OB neuronal connectivity was examined in a second model of CNG KO (Zhao and Reed 2001). This model exploited the expression of the CNG gene on the X chromosome to create a cellular mosaic in the olfactory epithelium that was comprised of two populations of sensory neurons, in heterozygous females. In one half of OSNs, ablation of the CNGA2 gene eliminated odor-evoked activity (CNGA2^−^). In the other half, the CNG gene was normally expressed (CNGA2^+^). This study reveals a dramatic loss of mutant OSNs in the olfactory epithelium. This loss was shown to be activity-dependent since OSNs expressing functional CNG channels survived, while OSN in which CNG expression was ablated, were lost. This activity-dependent competition was even more evident at the glomerular level, as CNG-deficient sensory axons innervate glomeruli along with wild-type OSN axons, but only CNG wild-type axons persisted, where CNG-deficient axons were gradually eliminated (Zhao and Reed 2001). The pruning of mitral cell dendritic tree was slowed down in CNG KO mice but ultimately results in a single apical dendrite, being mitral cells in KO mice indistinguishable from the ones in controls (Lin et al. 2000).

Mice homozygous for a null mutation in G_olf_ (Belluscio et al. 1998) exhibit a severe reduction in electrophysiological responses to a wide range of odor stimuli. Namely, the amplitude of electro-olfactogram (EOG) response to odors is reduced to 70–80% in comparison to odor response amplitude in controls. Despite the dramatic diminution in odor-evoked activity, OSN projections in stereotypic loci of the OB were unaltered. These results suggested a compensatory switch in the expression of G proteins in olfactory sensory cells. OSNs express two types of G protein, G_s_ and G_olf_. G_s_ is mostly expressed in the early phase of development, while G_olf_ is contained in mature OSNs. Genetic ablation of G_olf_ may trigger G_s_ expression, which is sufficient to ensure the stereotypical OSN projections and could account for the small residual EOG activity recorded.

Different results were observed upon the ablation of the adenylyl cyclase III (ACIII) gene, in OSNs. This genetic manipulation abolishes cAMP synthesis that in turn prevents the opening of CNG channels and the influx of Na^+^ and Ca^2+^ required to depolarize the membrane. Mice homozygous for a null mutation in ACIII show a complete loss of electro-olfactogram (EOG) response to odors. In addition, ACIII^−/−^ mutant mice were unable to detect lilial and citralva in the habituation behavioral test. Altogether, these data indicate that cAMP is essential in odor signaling. As for the projections of mutant sensory neurons, axons enter into the glomerular layer but do not coalesce to form discrete glomeruli, resulting in a deeply perturbed sensory map, in ACIII^−/−^ mice (Trinh and Storm 2003; Col et al. 2007) (Zou et al. 2007).

Altogether, these results indicate that odor-evoked activity, per se, does not have a major impact on sensory map formation. On the other hand, they corroborate the key role of the OR-derived cAMP in the coalescence of like axons to form glomeruli (see above). Indeed, among the genetic manipulations that interrupt the OR signaling pathway, only the mutation that hampers cAMP synthesis, i.e., ACIII ablation, resulted in a deeply perturbed sensory map. While OSNs project in stereotypic glomeruli in CNG and Golf mutant mice that bear genetic mutations that abolish odor-evoked activity but maintain unaltered cAMP synthesis.

## Spontaneous afferent activity refines and maintains specificity of synaptic connection in the olfactory bulb

The fact that odor-evoked activity is not required for the establishment of the highly ordered synaptic connections that define the olfactory map does not exclude the potential for the spontaneous afferent activity to play a role. OSNs exhibit spontaneous firing discharge, whose rate is different according to the OR expressed. In addition, OSNs expressing a defective OR, i.e., I7-RDY that is unable to couple G protein (see Imai et al. 2006) completely lack spontaneous activity, suggesting that spontaneous firing in OSNs is due to the spontaneous activation of G protein-coupled ORs (Connelly et al. 2013). These data indicate that the identity of ORs determines not only evoked but also spontaneous firing rate. However, basal activity exhibits high variability in OSNs expressing the same OR and there is a significant overlap in the firing rate of OSNs expressing different ORs (Connelly et al. 2013) (Reisert 2010). The spontaneous conformational change of ORs prompted to hypothesize that ligand-independent activation of ORs could be at the origin of the OR-derived cAMP, which is required to direct OSNs to invariant locations in the OB (Nakashima et al. 2013). The vast diversity of ORs, the high variability of cAMP levels even among OSNs expressing the same OR makes unclear how specificity can be achieved according to this model.

Spontaneous and evoked activity, although originate both from ORs, seem to be independently generated. Basal activity is not significantly affected in CNG (Brunet et al. 1996), ACIII (Wong et al. 2000), and Golf (Belluscio et al. 1998) mutants. This corroborates the potential for a role of spontaneous activity in the formation of the sensory map.

The impact of afferent spontaneous discharge on the sensory map formation was analyzed in distinct genetic mouse models. In one model, conditional expression of the light chain of tetanus toxin, a molecule that inhibits neurotransmitter release, did not affect the convergence of OSNs to form glomeruli in invariant locations in the OB (Yu et al. 2004). When the expression of the light chain of tetanus toxin was restricted to a subpopulation of OSNs, e.g., P2-OR expressing neurons, resulting in inhibition of neurotransmission only in this subpopulation of neurons, the mutant axons exhibited a progressive mistargeting behavior and disappeared. These data indicate that, under competitive conditions, neurons defective in neurotransmission, project to a glomerular locus, but fail to maintain synaptic contacts and reroute to inappropriate targets throughout the bulb. The inability to maintain stable synaptic connections is followed by a striking reduction in sensory neurons (Yu et al. 2004).

In a second mouse model, conditional overexpression of the inward rectifying potassium channel Kir 2.1, results in hyperpolarization OSNs and causes a dramatic reduction of their basal firing rate (Yu et al. 2004). A thorough and detailed analysis of the electrophysiological properties of Kir2.1 overexpressing OSNs demonstrated that spontaneous discharge was strikingly reduced, while odor-evoked responses remained unaffected in Kir2.1 overexpressing OSNs in respect to controls (Lorenzon et al. 2015). The selective impairment of spontaneous activity makes the transgenic line of Kir2.1 mice a unique useful model to dissect the impact of spontaneous discharge on axon targeting. OSNs overexpressing Kir2.1 enter the bulb with significant delay. Analysis of the sensory map unraveled a deeply disrupted organization of glomeruli in the dorsal aspect of the bulb. This region remained thinned innervated, with glomeruli of giant dimension and absence of neckless glomeruli. By studying the projections of axons expressing specific OR in Kir2.1 mice, it was shown that P2 expressing neurons targeted not only the main glomerulus on the medial and another main glomerulus on the lateral side of each bulb but also several additional glomeruli. The sensory map resulted therefore deeply disrupted in Kir 2.1 mice (Yu et al. 2004). A more detailed and thorough analysis of the consequences of the absence of spontaneous afferent activity on neuronal wiring in the OB (Lorenzon et al. 2015) revealed that in Kir2.1 mice, additional glomeruli were heterogeneous in their organization, e.g., formed by axons expressing different ORs, and were located in a circumscribed areas around the main homogeneous glomeruli. The defects in connectivity in the OB of Kir2.1 mice were mirrored with exquisite precision in aberrant features of the functional maps recorded in the OB and in impairments in odor discrimination behavior, in Kir2.1 mice. Lorenzon and collaborators extended the analysis of the impact of spontaneous afferent activity also to the second level of topography in the olfactory bulb: the link between homologous glomeruli. First, they characterized the development of the link between homologous glomeruli, since previous studies were limited to adults (Belluscio et al. 2002; Lodovichi et al. 2003). They found that the link in the early phase of development (postnatal day 7) is not confined to a single glomerulus but larger. Through a refinement process, ETC projection becomes restricted to a single glomerulus (at postnatal day 30), providing a point-to-point connection between homologous glomeruli. Analysis of connectivity between homologous glomeruli revealed that the link was preserved in absence of spontaneous afferent activity, but the projections of ETC were not confined to the homologous glomerulus, but larger in adult Kir2.1 mice than in controls. The morphology of ETC projection in Kir.2.1 mice resembles the one present in the early stages of development, in controls (Lorenzon et al. 2015). Altogether, these data indicate that in absence of spontaneous afferent activity the olfactory map and the intrabulbar link are established, but neuronal connectivity within these structures remains unrefined, assuming morphological features proper of immature neuronal circuits. This unrefined connectivity, in turn, impairs the functional outcome of OB networks and olfactory discrimination behavior.

Neuronal circuits in the brain are shaped by experience during “critical periods,” i.e., epochs of heightened brain plasticity, in early postnatal life. Critical periods have been thoroughly dissected in other sensory modalities, such as the visual system and somatosensory system (Hensch 2004). Whether a critical period exists also in olfaction remains largely to be defined. By exploiting the inducible nature of the Kir2.1 construct, Lorenzon et al. (2015) revealed that overexpression of Kir2.1 in adult OSNs, once glomeruli are already properly formed, induced a rerouting of OSN axons away from the main homogenous glomeruli. These wandering axons targeted additional glomeruli located in the vicinity of the main glomerulus, which resulted heterogeneous in their organization. Similar results were observed in the connectivity of the intrabulbar link. Overexpression of Kir2.1 in adults induced regression of the already refined ETC projection. These data provide compelling evidence that neuronal circuits in the OB maintain a high degree of plasticity throughout adulthood. These findings are in line with previous evidence that supports lifelong plasticity in the OB (Gogos et al. 2000). Other works (Ma et al. 2014; Tsai and Barnea 2014), using genetic approaches to express proteins that can interfere with OSN axons targeting, seem to have identified an unconventional critical period around the first week of postnatal life, beyond which the persistence of genetic manipulation lead to permanent alterations of OSNs targeting. The constant neurogenesis of OSNs, that might hold a key to plasticity throughout life, and the mixed evidence related to an unconventional critical period, highlight the need for further investigations to clarify the extent and features of a critical period in the olfactory system.

## Conclusions and perspectives

Here, I provided an overview of the mechanisms underlying the formation of olfactory neuronal circuits, with a focus on recent findings on the role of the odorant receptors and spontaneous afferent activity in neuronal wiring, in mice.

The specificity of connections in olfactory neuronal circuits provides a discrete topographic organization of the OB, which emerges from an almost random distribution of sensory neurons in the olfactory epithelium. The discrete nature of the OB organizational plan hinges on the OR identity, which defines not only the molecular receptive range of OSNs but also their target in the brain. Recent studies have elucidated how the OR can accomplish such a dual role, beginning to decipher the mechanism of activation of the axonal OR and unraveling its putative ligands. This solved a long-standing enigma in the field and opened a new chapter of investigations, aimed at identifying additional putative ligands for axonal ORs. It emerged that the OR identity is associated and can regulate the expression of other molecular determinants. Furthermore, ORs can dictate not only the evoked but also the spontaneous discharge of OSNs, which was reported to contribute to maintaining and refining neuronal circuits.

Although consistent knowledge has been acquired on the mechanism underpinning the formation of neuronal circuits that provide the olfactory map of the OB, the logic underpinning the link between homologous glomeruli remains largely to be defined. Since ETC projection links glomeruli that express the same OR, it is tempting to speculate that gradients of molecules whose expression is related to ORs direct also the axonal projection of ETC. Whether the molecular determinants are the same or different in respect to the ones that direct OSNs to their glomerular target remains to be clarified.

The dual role of odorant receptors, i.e. detecting odors and directing OSN axons to their targets in the brain, unveils an elegant mechanism by which Nature solved the challenging feat of creating spatial order among more than one thousand ORs, to create a topographic map that encodes a wide range of odorant stimuli.
